# MRI Detects Brain Reorganization after Human Umbilical Tissue-Derived Cells (hUTC) Treatment of Stroke in Rat

**DOI:** 10.1371/journal.pone.0042845

**Published:** 2012-08-10

**Authors:** Quan Jiang, Christine Thiffault, Brian C. Kramer, Guang Liang Ding, Li Zhang, Siamak P. Nejad-Davarani, Lian Li, Ali S. Arbab, Mei Lu, Brad Navia, Stephen J. Victor, Klaudyne Hong, Qing Jiang Li, Shi Yang Wang, Yi Li, Michael Chopp

**Affiliations:** 1 Department of Neurology, Henry Ford Health System, Detroit, Michigan, United States of America; 2 Department of Public Health Sciences, Henry Ford Health System, Detroit, Michigan, United States of America; 3 Department of Radiology, Henry Ford Health System, Detroit, Michigan, United States of America; 4 Advanced Technologies and Regenerative Medicine, LLC, a Johnson & Johnson Company, Somerville, New Jersey, United States of America; 5 Johnson & Johnson Pharmaceutical Research and Development, Titusville, New Jersey, United States of America; 6 Department of Physics, Oakland University, Rochester, Michigan, United States of America; Hôpital Robert Debré, France

## Abstract

Human umbilical tissue-derived cells (hUTC) represent an attractive cell source and a potential technology for neurorestoration and improvement of functional outcomes following stroke. Male Wistar rats were subjected to a transient middle cerebral artery occlusion (tMCAo) and were intravenously administered hUTC (N = 11) or vehicle (N = 10) 48 hrs after stroke. White matter and vascular reorganization was monitored over a 12-week period using MRI and histopathology. MRI results were correlated with neurological functional and histology outcomes to demonstrate that MRI can be a useful tool to measure structural recovery after stroke. MRI revealed a significant reduction in the ventricular volume expansion and improvement in cerebral blood flow (CBF) in the hUTC treated group compared to vehicle treated group. Treatment with hUTC resulted in histological and functional improvements as evidenced by enhanced expression of vWF and synaptophysin, and improved outcomes on behavioral tests. Significant correlations were detected between MRI ventricular volumes and histological lesion volume as well as number of apoptotic cells. A positive correlation was also observed between MRI CBF or cerebral blood volume (CBV) and histological synaptic density. Neurological functional tests were also significantly correlated with MRI ventricular volume and CBV. Our data demonstrated that MRI measurements can detect the effect of hUTC therapy on the brain reorganization and exhibited positive correlation with histological measurements of brain structural changes and functional behavioral tests after stroke. MRI ventricular volumes provided the most sensitive index in monitoring brain remodeling and treatment effects and highly correlated with histological and functional measurements.

## Introduction

Treatment of ischemic stroke is currently restricted to acute thrombolytic drugs, which are only effective if administered within 4.5 hours of the ischemic event [1], [2]. In practice, fewer than 5% of the ischemic stroke patients are treated due to the very narrow treatment window and other exclusion criteria [1]. Disabilities resulting from stroke are further compounded by the lack of treatments that can be given days to weeks after stroke onset to promote functional recovery. Experimental studies in rodent stroke models suggest that cell-based neurorestorative treatments enhance brain reorganization and substantially improve functional recovery when treatment is initiated days or up to one month after stroke [3], [4]. Neurorestorative treatments amplify endogenous processes of brain plasticity, including vascular and neuronal remodeling which likely contribute to improvement in neurological function after stroke [5]. Current understanding of vascular and neuronal remodeling after stroke, however, has been derived mainly from *post mortem* regional measurements of stained cerebral tissue sections using histological and immunohistological methods [5]. As such, analysis is restricted to a single (terminal) time point which does not allow for dynamic assessment of tissue remodeling. In contrast, MRI can noninvasively monitor the temporal profiles of functional recovery and tissue remodeling after stroke [6], [7], [8]. The traditional MRI method for monitoring stroke recovery is functional MRI (fMRI) [8]. However, fMRI is based on hemodynamic changes in blood oxygen extraction rate, cerebral blood flow (CBF), and cerebral blood volume (CBV) [9]. Although fMRI provides crucial information related to recovery after stroke, fMRI does not provide data related to changes in brain structure after stroke. We have demonstrated that MRI can monitor the structural changes of vascular and neuronal reorganization in preclinical models of stroke [6], [7], [10]. However, the correlations between functional neurological outcome and a complete set of MRI measurements of brain neuronal and vascular structure histological changes have not yet been evaluated. Due to the non-invasive nature of MRI measurement, the MRI indices of vascular and neuronal remodeling related to neurological outcome in preclinical stroke models has the potential to be translated to clinic.

Intravenous administration of human umbilical tissue-derived cells (hUTC) has been shown to improve functional and histological outcomes (synaptic and vessel density) in young adult rats in a temporal middle cerebral artery occlusion (tMCAo) model of ischemic stroke [11]. In the present study, we investigated the ability of a complete set of MRI measurements to detect vascular and white matter remodeling in tMCAo following the administration of human umbilical tissue-derived cells (hUTC). Furthermore, we attempted to correlate MRI measurements obtained over time with either neurological functional outcomes (modified neurological severity score (mNSS), adhesive removal and foot-fault test) or terminal histological outcomes (myelin, axon, apoptosis, synaptic and vessel densities) obtained from the same subjects.

## Results

### Quantitative MRI Measurements

**Figure 1:**
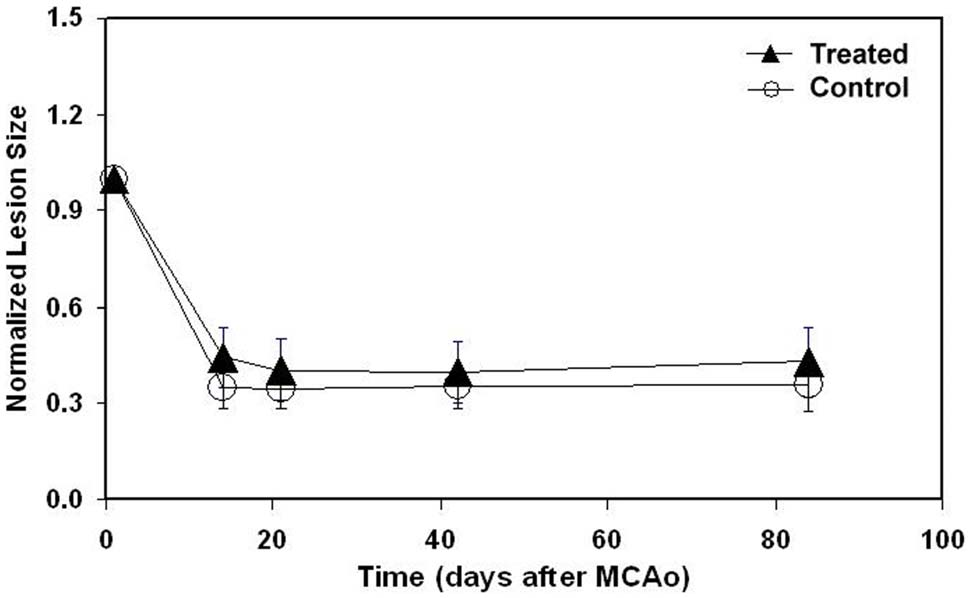
The temporal profiles of lesion volumes. The temporal profiles of relative lesion volumes in tMCAo rats model following the administration of hUTC or vehicle.

The relative ischemic damaged volumes were measured in the lesion using T_2_ maps at 24 hrs, 2, 3, 6 and 12 weeks post-tMCAo and normalized to the lesion volume obtained at 24 hrs after tMCAo to reduce individual variability. No treatment-by-time interaction was detected on the relative ischemic damaged volume (p = 0.24), indicating that the treatment effects on ischemic volume were consistent over time. The relative ischemic damaged volumes were significantly decreased (p<0.05, Fig. 1) over time compared to that obtained at 24 hrs in both control and hUTC groups, however there was no hUTC treatment effect on the relative ischemic volume, compared to controls.

**Figure 2:**
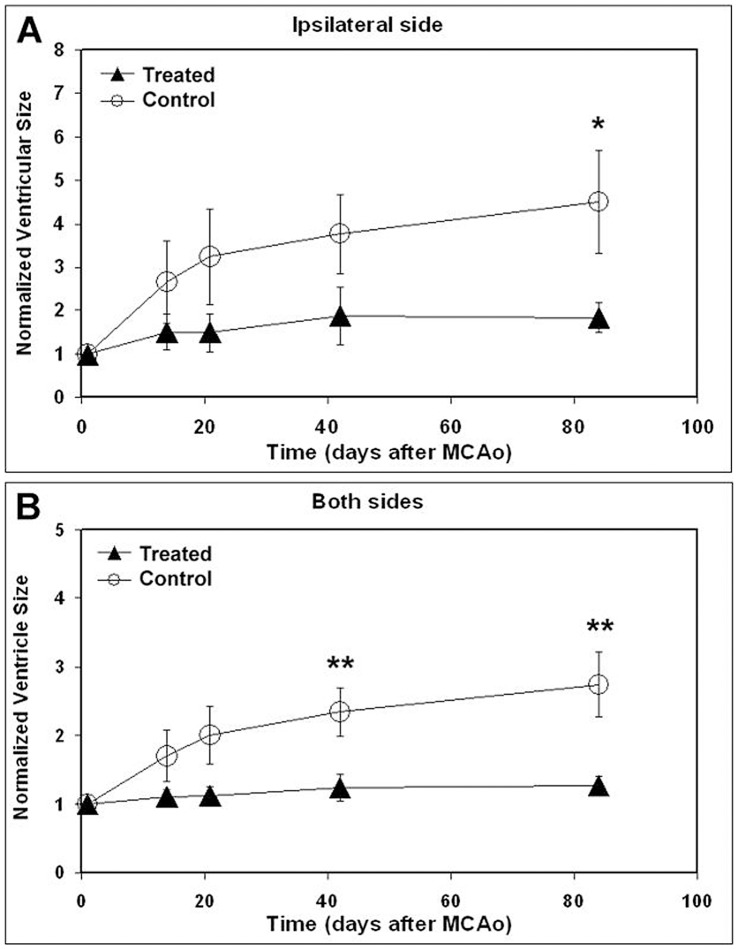
The temporal profiles of ventricle volumes. The temporal profiles of relative ventricle volumes in ipsilateral hemisphere (A) and bilateral hemisphere (B) of hUTC or vehicle treated groups, respectively. *, p<0.05, control vs treated groups; **, p<0.01, control vs treated groups.

The relative ventricular volumes were measured using T_2_ maps at 24 hrs, 2, 3, 6 and 12 weeks post-tMCAo and normalized to the ventricular volume obtained at 24 hrs to reduce individual variability. Normalization of ventricular volumes should also be performed relative to prestroke conditions. The relative ventricular volume measured in the ipsilateral hemisphere (Fig. 2A) was significantly lower in hUTC treated group, compared to vehicle controls at week 12 (p = 0.029). The relative ventricular volume in the whole brain (both hemispheres, Fig. 2B) was significantly lower in hUTC treated group compared to vehicle treated group at week 6 and week 12, respectively. In addition, in the vehicle treated group, the relative ventricular volume in either ipsilateral or bilateral hemispheres was significantly increased over time during the 12-week period compared to the volume measured at 24 hrs. In contrast, a similar increase in the ventricular volume was not observed over time in the brain of hUTC treated animals.

**Figure 3:**
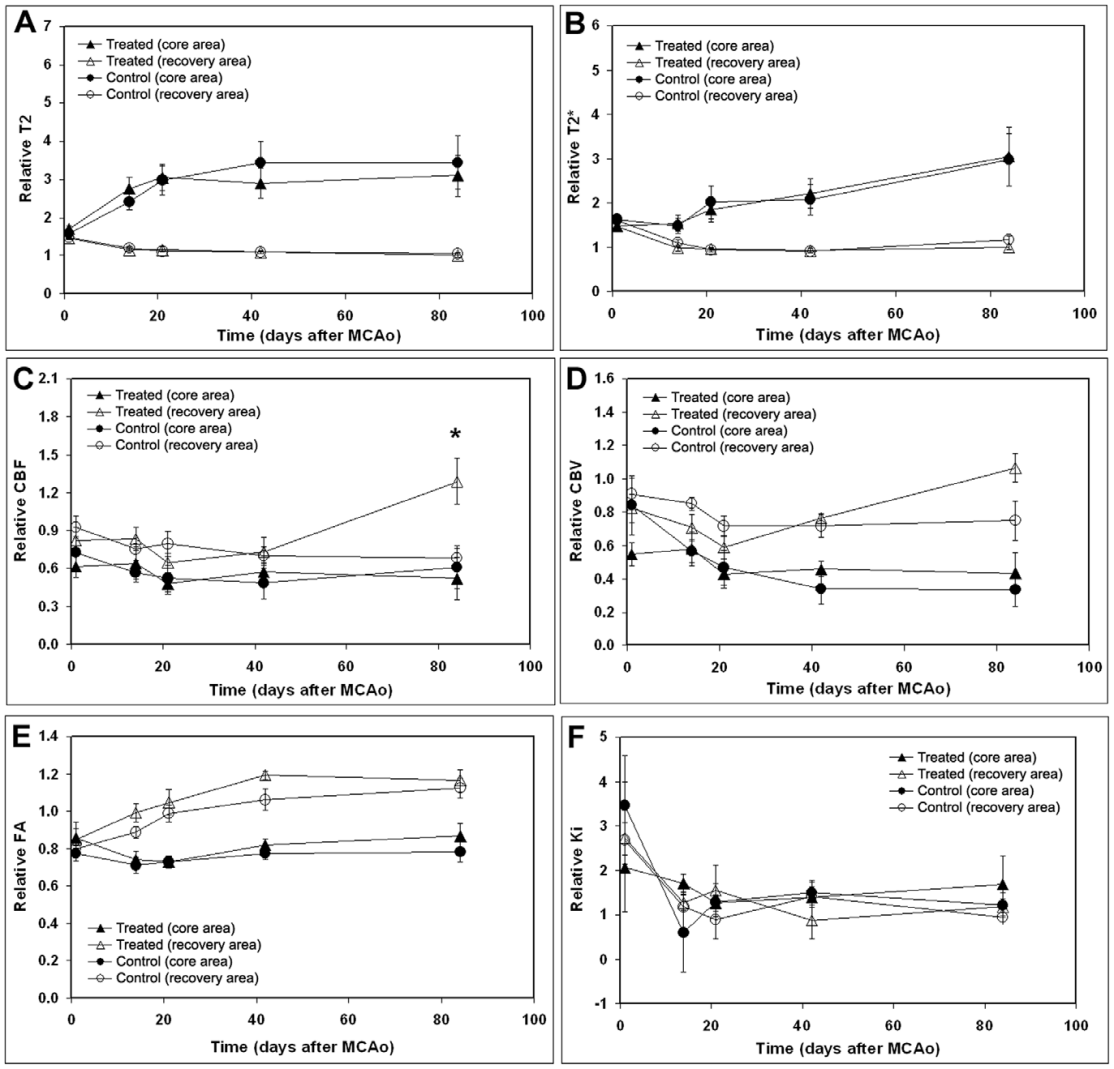
The temporal profiles of T_2_, T_2_*, CBF, CBV, FA, and K_i._ The temporal profiles of relative T_2_ (A), T_2_* (B), CBF (C), CBV (D), FA (E), and K_i_ (F) values for the ischemic recovery (recover) and ischemic core (core) ROIs in hUTC or vehicle treated animals, respectively. *, p<0.05, control vs treated groups.

Measurements of T_2_, T_2_*, CBF, CBV, FA, and K_i_ were performed on the ischemic core and recovery ROIs. The relative changes of MRI measurements in the ischemic core ROI (ischemic core/contralateral core) and recovery ROI (ischemic recovery/contralateral recovery) were calculated and used in the analysis. Figures 3A–F illustrate temporal profiles of relative T_2_ (Fig. 3A), T_2_* (Fig. 3B), CBF (Fig. 3C), CBV (Fig. 3D), FA (Fig. 3E), and K_i_ (Fig. 3F) from the ischemic core and recovery ROIs in the brain of both hUTC and vehicle treated groups after stroke. The relative T_2,_ and T_2_* exhibited similar temporal profiles (Fig. 3A–3B) in both hUTC and vehicle treated groups. The mean values of T_2,_ and T_2_* in the ipsilateral ischemic core ROI were higher than those in the homologous contralateral ROIs (>1, Fig. 3A–3B) at 24 hrs after stroke in both groups. The relative T_2,_ and T_2_* remained significantly lower in the recovery ROI compared to the ischemic core ROI from week 2 to week 12 after stroke. The differences in the two ROIs were consistent across treatments and time. However, hUTC treatment had no significant effect on relative T_2_ or T_2_*.

Although the relative CBF and CBV remained low in the ischemic core ROI compared to the recovery ROI from 2 to 12 weeks after tMCAo, significant differences (p<0.01) between the two ROIs were only detected at week 6 (CBV) and 12 (CBV and CBF) in hUTC treated group and at week 2 (CBV) and 3 (CBV and CBF) in the vehicle treated group. In addition, hUTC treatment significantly improved CBF (p<0.05) and marginally improved CBV (p = 0.054) in the recovery area at 12 weeks post-tMCAo, compared to controls.

FA values were significantly different between the two regions of ischemic core and recovery (p<0.01), which did not depend on either treatment or time. The mean relative FA values (Fig. 3E) in the ischemic core remained low (<1) during the course of this study (12 weeks) in both control and treated groups. However, the mean relative FA values in the ischemic recovery ROIs gradually increased (p<0.05) from week 2 to 12 in both control and treated groups. The mean relative FA values obtained from animals administered hUTC were higher in the ischemic recovery regions compared to the control group; however, it did not reach statistical significance.

The mean values of K_i_ in both ischemic core and recovery ROIs were higher than those obtained in the homologous contralateral ROIs (>1, Fig. 3F) at 24 h after stroke (before treatment administration). There was no significant difference in K_i_ measurement between hUTC and vehicle animals in either the ischemic core or recovery ROIs from 2 to 12 weeks after tMCAo.

**Figure 4:**
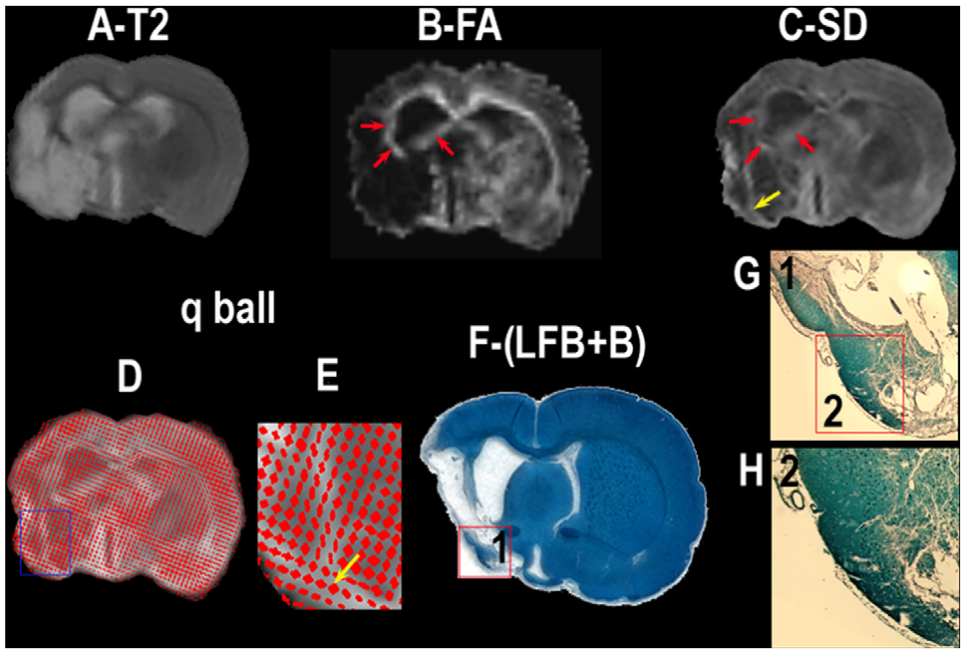
MRI detection of white matter reorganization after hUTC treatment of stroke. Ex vivo T2 (A), FA (B), SD (C), q-ball fiber orientation map (D, E), and Bielshowsky and Luxol fast blue immunoreactive staining images (F–H) measured in the fixed animal brain. High-magnification q-ball (E) or Bielshowsky and Luxol fast blue (G, H) images from the areas shown in the box in the q-ball (D) or Bielshowsky and Luxol fast blue (F, G) as indicated in the top right-hand corners of (E, G, H).

*Ex vivo* FA and diffusion standard deviation (SD) measurements were performed 12 weeks after stroke on post-mortem unfixed brain tissues to confirm *in vivo* MRI findings and to compare the differences between traditional DTI measurement, FA, with Q-space DTI measurement of SD. Figure 4 shows *ex vivo* T2 (A), FA (B), SD (C), q-ball fiber orientation direction overlaid to SD map (D, E), and Bielshowsky and Luxol fast blue images (F–H) from a representative hUTC treated rat brain 12 weeks after stroke. White matter reorganization, confirmed by an increase in axons (G–H, black) and myelination G–H, blue), coincided with increases in FA (B, red arrows) in the extended region of the corpus callosum and dentate gyrus surrounding the lesion. The SD map revealed increased SD not only at the boundary of the lesion (C, red arrows) as shown on the FA map but also in the piriform cortex area of the lesion (yellow arrow), where fiber crossings of axons were confirmed by the q-ball fiber orientation map (E, yellow arrow) and the Bielshowsky and Luxol fast blue images (G, H). Statistical analysis was performed on FA and SD data between treated and control groups. The mean relative values of FA and SD in the recovery ROI were higher in the treated group (FA, 1.05±0.25; SD, 1.23±0.31) compared to the control (FA, 0.98±0.24; SD, 1.04±0.10) rats. However, there were no significant differences whether FA and SD were measured *ex vivo* or *in vivo*, confirming the validity of the *in vivo* findings.

### Quantitative Histological Measurements

The quantitative *ex vivo* histological findings obtained from the brain of rats administered hUTC or vehicle are summarized in Table 1. No significant difference in the lesion volume, number of apoptotic cells (TUNEL staining), or percent of axon and myelin volume (Bielschowsky and Luxol fast blue staining) could be detected among the groups. The level of vWF expression was significantly (p = 0.0072) higher in the brain of hUTC treated rats compared to the control group at 12 weeks after tMCAo. The level of synaptophysin expression was also significantly (p = 0.0103) higher in the IBZ of hUTC-treated animals compared to control animals.

**Table 1:** Histological measurements: Mean ± SD.

Conditions	Control	Treated
% Lesion volume (a)	26.66±17.18 (10)	26.68±15.19 (11)
vWFpositive vessels/mm2 (b)	192.76±42.65 (10)	243.90±43.45 (11)**
% Synaptophysin positive area (c)	11.23±1.18 (10)	14.36±4.30 (11)*
TUNEL cells/mm2 (d)	39.29±18.52 (10)	20.37±11.55 (9)
Bielschowski and luxol Fast Blue (e)	104.56±14.60 (10)	110.95±18.43 (11)

N =  number of rats/group is indicated in the brackets.

(a) Lesion volume is expressed in percent compared to the contralateral intact section of the brain.

(b) von Willebrand factor (vWF) is a marker for blood vessels. Density of vWF vessels in IBZ (vessels/mm^2^).

(c) Synaptophysin, a marker of synapses, is expressed in % of cells in the IBZ.

(d) Apoptosis is expressed as the number of apoptotic cells per mm^2^.

(e) Bielschowski and luxol Fast Blue are markers of axon and myelin, respectively, and indicate changes in axonal myelination. Data is represented as % of contralateral intact IBZ.

* =  significant differences, p<0.05;

** =  significant differences, p<0.01.

### Quantitative Functional Test Measurements

**Figure 5:**
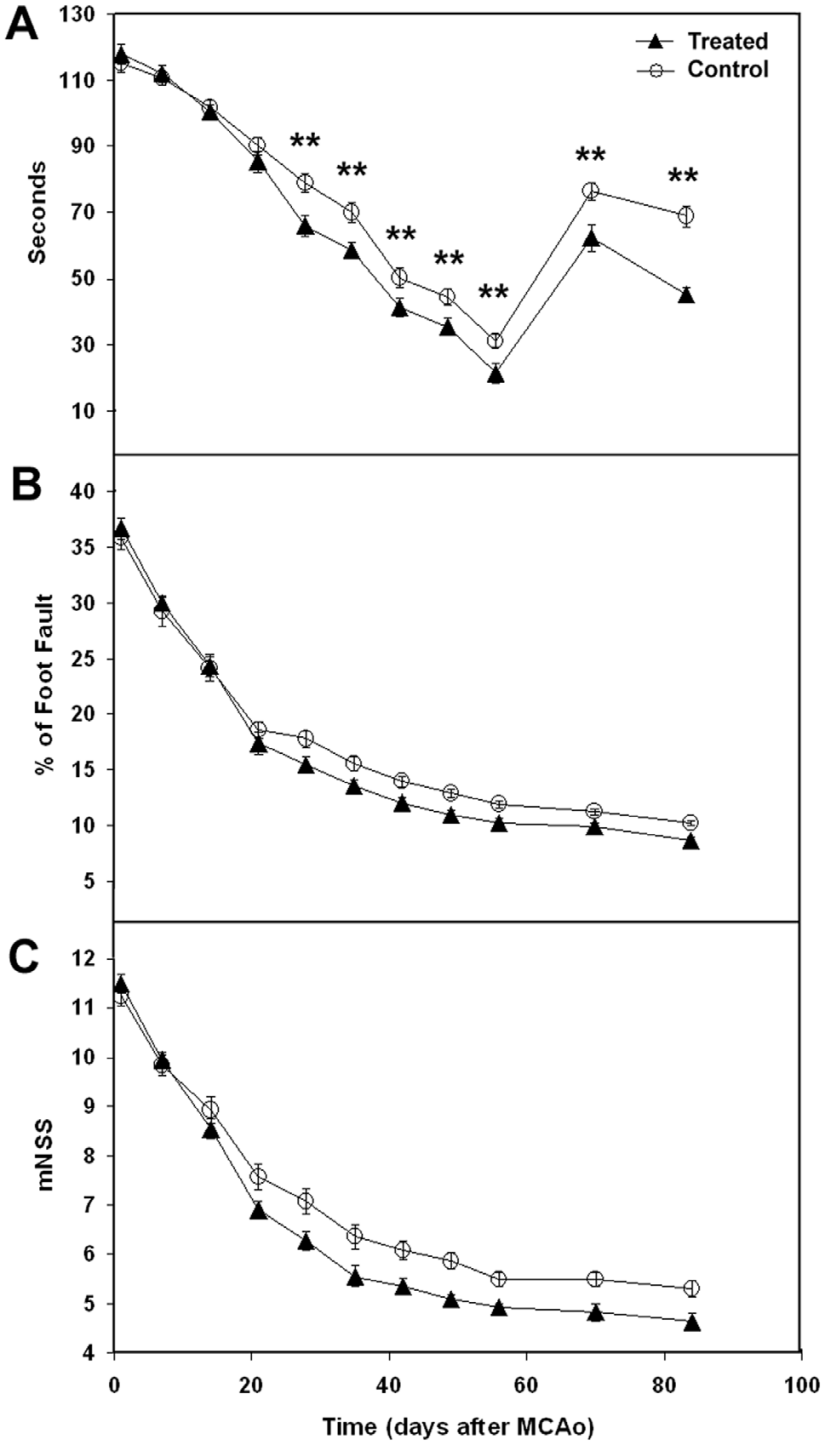
The temporal evolution of functional neurobehavioral tests. The temporal evolution of functional adhesive removal (A), foot-fault (B), and mNSS (C) neurobehavioral tests, respectively, obtained at various times from 1 day to 12 weeks after stroke. **, p<0.01, control vs treated groups based on the global test.

Functional behavioral tests (mNSS, adhesive removal test and foot-fault test) were performed on animals administered either 111-Labeled hUTC or vehicle (control) starting at one day post-tMCAo and weekly thereafter from 1 to 12 weeks after tMCAo. No significant difference in the behavioral score obtained from each functional assay could be detected at 1 day after stroke (baseline before treatment) between the two treatment groups (Fig. **5**). Rats treated with hUTC exhibited significant functional recovery at weeks 4 to 12 compared to the control treated group (Fig. **5**).

### Correlation between MRI and Histological Measurements

Histological lesion volume was significantly correlated with MRI lesion volume (r = 0.90596, p<0.0001), ventricular volumes measured bilaterally (r = 0.50366, p = 0.0053), and ipsilaterally (r = 0.54993, p = 0.0020), relative T2* (r = 0.69342, p<0.0001) and T2 (r = 0.59812, p = 0.0005) in ischemic core, and subtractions of T2* between ischemic core and recovery ROIs (T2*_core_-T2*_recovery_; r = 0.68129, p<0.0001) and T2 (T2_core_-T2_recovery_; r = 0.56838, p = 0.0010).

The number of apoptotic cells (TUNEL) was significantly correlated with the ventricular volumes measured bilaterally (r = 0.71290, p<0.0001) and ipsilaterally (r = 0.69870, p<0.0001), relative T2 (r = 0.61256, p = 0.0003) in the ischemic core, and for subtractions of T2 between ischemic core and recovery ROIs (T2_core_-T2_recovery_; r = 0.60416, p = 0.0004).

The synaptic density was positively correlated with the difference between CBV in the ischemic core and recovery ROIs (CBV_recovery_- CBV_core_; r = 0.51979, p = 0.0065). Whereas synaptic density positively correlated with CBF in both ischemic core (r = 0.51419, p = 0.0086) and recovery (r = 0.79517, p<0.0001) ROIs.

### Correlation between MRI, Functional and Histological Measurements

The percent of foot-faults was significantly correlated with the ventricular volume measured in both hemispheres at week 12 (r = 0.50774, p = 0.0069). The improvement in foot-fault measurement was also significantly correlated with the ventricular volume measured in the ipsilateral hemisphere at week 12 (r = 0.54652, p = 0.0032). The neurobehavioral (mNSS test) improvement was significantly correlated with the lesion volume as measured by MRI at week 12 (r = 0.43574, p = 0.0205). The time required to removed the adhesive paper was significantly correlated with CBV in the ischemic recovery ROI (r = 0.41847, p = 0.0334) and was marginally correlated with FA in the ischemic recovery area (r = 0.36247, p = 0.0580).

## Discussion

In this study, MRI was shown to be a useful imaging tool to detect evidence of brain reorganization following the administration of 111-In labeled hUTC. Animals treated with 111-In labeled hUTC showed significantly enhanced expression of vWR and synaptophysin, and improved functional recovery, similar to results obtained with unlabeled hUTC^11^. These results suggest that the cells used here are similar to unlabeled hUTC used previously. In addition, functional improvements were significantly correlated to reduction of ventricular volume expansion and improvement of CBV measured by MRI. Furthermore, ventricular volume, as measured by MRI, provided the most sensitive index of brain structure reorganization. Ventricular expansion, a structural sequela of brain injury, reflects global atrophy of the brain [12], [13], [14], [15]. In the clinic, most stroke patients show cerebral atrophy in the chronic stage of stroke [14], [15]. Severe ischemic damage could cause increases in the lesion volume, expansion of the ventricles, increases in the number of apoptotic cells, and a global atrophy of the brain. Therefore, ventricular volume as monitored by MRI can be used as a marker of the severity of the ischemic damage. This is demonstrated by the positive correlation observed between the ventricular volume (MRI) with the lesion volume and the number of apoptotic cells (TUNEL) in the ischemic lesion. Reduction of ventricular expansion may be related to improvements in tissue structure as evidenced by increased vessel and synaptic densities. In fact, others have reported increased angiogenesis, axonal integrity and synaptogenesis following neurorestorative therapy in stroke models [16], [17]. Altogether these changes can act to improve tissue structures in the ipsilateral hemisphere, including the ventricular boundary.

Data presented in this study also revealed significant correlations between MRI measurements of T_2_, T_2_* and histological measurements of lesion volume and apoptotic cells in a manner that is consistent with tissue injury. It is known that T_2_ and T_2_* increase with the water content and tend to decrease with complex tissue structures. Severe ischemic damage not only causes an increase in the lesion volume and apoptotic cells but also adversely affects the structure of the brain (e.g. alters extracellular matrix, reduces axonal and synaptic densities, and increases water content), which is reflected by the increase in T_2_ and T_2_* measurements in the ischemic core.

We demonstrated that animals treated with hUTC had higher expression of vWF and restored CBF and CBV, which corresponded with improved neurological function. Cell-based treatments have been shown to amplify endogenous processes of vascular remodeling such as angiogenesis which likely contribute to the improvement in neurological function after stroke [18], [19], [20]. Higher cerebral blood vessel density predicts improved outcome and survival in stroke patients [21], [22], and neurorestorative treatments have been shown to induce angiogenesis [23], [24], [25], [26], [27], [28]. In rats, neurorestorative therapy of stroke increases levels of vascular endothelial growth factor (VEGF) [24],[29],[30],[31],[32], which enhances vessel density and arteriogenesis primarily in the boundary zone of the ischemic lesion, and reduces functional deficits [24], [29], [30], [31], [32] These newly formed vessels are important for tissue perfusion, but most likely their benefit derives from the factors expressed by newly formed vessels, such as BDNF, VEGF, VEGFR2 and matrix metalloproteinases (MMP) like MMP 2 and MMP 9 [33], [34]. The current data are consistent with previous investigations showing that cell-based treatment increases vessel density and restores CBF and CBV, which parallel the improvement in neurological functional performance [7], [35], [36]. While, MRI CBF and CBV did not significantly correlate with histological measurement of vessel density (vWF), they do, highly correlate with higher expression of synaptophysin. The reasons for these complex relationships are presently unknown. However, they may be related to the differences of data analysis between MRI and histology and the local changes between vascular and synaptic densities. Although the MRI ROI analysis method utilized in this study is reliable and reproducible, the sensitivity is relatively low due to averaging of a small area of vascular remodeling over the larger recovery area. In addition, the histological measurements are taken from a small area with large regional variation in microvessel density compared to the recovery area captured by MRI due to field of view limitations which could obscure the correlation between vascular density and CBF and CBV derived by MRI. In contrast, there was low regional variation in synaptic density measured by histology which correlated with MRI CBF and CBV. Furthermore, others have reported that vascular remodeling was linked to synaptogenesis following neurorestoration in rodent models of stroke [25], [37], [38], [39], [40]. Cerebral blood vessels mainly provide nutritive blood flow and cerebral endothelial cells secrete factors which could promote neurogenesis and synaptogenesis as discussed above [25], [37], [38], [39], [40]. Accordingly, the interaction between synaptogenesis and vascular remodeling may contribute to the positive correlation between MRI CBF and CBV and synaptogenesis as measured by histology.

Significant correlations were detected between MRI assessment of ventricular volume and neurological foot-fault test, MRI lesion volume and mNSS test, as well as MRI CBV, while marginal (marginal, p = 0.058) correlations were detected between FA and adhesive-removal test. It is known that these neurological tests are sensitive measures of functional recovery after restorative treatment of stroke in rats [5], [37], [38], [41]. To the best of our knowledge, this is the first systematic investigation evaluating the correlations between a complete set of MRI measurements of vascular and neuronal structural changes, histological measurements of vWF and synaptophysin and neurological tests. The direct relationship between the functional tests and the individual modifications of tissue may be difficult to identify. It is possible that beneficial treatment effects are derived from the interaction between neuronal and vascular remodeling, since overall brain functional recovery depends on synergistic interactions between multiple events. MRI ventricular volume was able to detect hUTC effects and significantly correlated with the neurological foot-fault test. Neurological foot-fault test is known to detect limb motor coordination deficits. The improved tissue structures, as indicated by MRI ventricular volume, could also promote the reestablishment of axonal connections from the cortical spinal track to motor cortex [6], [42]. Although there are no statistically significant differences in axonal density measured by Bielschowsky and luxol fast blue staining, the mean values of FA, and SD were higher in the hUTC group compared with those in the control animals. The functional improvements measured by neurological foot-fault test may be caused by the reestablishment of axonal connections from a few axonal bundles which could not be detected using the histological techniques and MRI measurements. As stated above, our current MRI analysis may not be sufficiently sensitive to detect small differences in brain structure. Our data also did not demonstrate an advantage of SD over FA in detecting axonal remodeling. FA is a traditional MRI measure of axonal changes. However, an overall artifactual lowering of FA occurs when white matter fiber tracts cross due to the assumption of Gaussian diffusion tensor model [43]. Q-space diffusion tensor imaging (q-DTI), such as SD can better detect the axonal remodeling, especially during the early stage of axonal remodeling which involves less organized, random oriented axons [44]. The reasons for the relatively equivalent sensitivity in detecting axonal remodeling between FA and SD may be attributed to the time of measurements. The reduced differences in FA and SD may be reflective of the reorganization of axonal bundles taking place at 12 weeks after stroke into mature and well organized fibers into a single direction [42].

Our results also demonstrated that the neurological mNSS test significantly correlated with MRI lesion volume. Neurological mNSS test involves motor, sensory, balance, and reflex functions [41]. Lesion volume also affects the overall performances of stroked animal. However, the mechanisms why each neurological functional behavioral test correlated to specific MRI measurements are unknown and need to be further investigated.

In conclusion, we demonstrated that MRI measurements can detect the effect of cell-based therapy on the brain reorganization and exhibited positive correlation with histological measurements of brain structural changes and functional behavioral tests after stroke. MRI ventricular volumes provided the most sensitive index in monitoring brain remodeling and hUTC effects and highly correlated with histological and functional measurements. Due to the non-invasive nature of MRI measurement, the MRI indices of brain remodeling related to neurological outcome in this preclinical stroke study could be translated to clinic.

## Materials and Methods

All experimental procedures have been approved by the Institutional Animal Care and Use Committee and Institutional Review Board of Henry Ford Hospital. All efforts were made to ameliorate suffering of animals.

### Collection and Preservation of Human Umbilical Tissue Derived Cells (hUTC)

hUTC were isolated and expanded from the umbilical cord of a single donor under full consent as previously described [11]. Briefly, the umbilical cord was manually minced and digested with a mixture of 0.5 Units/ml collagenase (Nordmark, Uetersen, Germany), 5.0 Units/ml, dispase (Roche Diagnostics, Indianapolis IN), and 2 Units/ml hyaluronidase (ISTA Pharmaceuticals, Irvine CA) until almost completely digested. The cell suspension was passed through a sieve to remove undigested tissue. Harvested cells were grown to approximately 5–7 population doublings (PD) on standard tissue culture flaskes. The cells were then cultured on Hillex II microcarriers (SoloHill Engineering, Ann Arbor MI) in bioreactor containing Growth Media (DMEM-low glucose, 15% (vol/vol) defined fetal bovine serum (FBS; HyClone, Logan UT), with 100 Units/ml penicillin, and 100 µg/ml streptomycin (Invitrogen, Carlsbad CA). The cells were cultured under standard conditions in atmospheric oxygen with 5% carbon dioxide at 37°C and allowed to culture to approximately 25–30 total cumulative population doublings. Finally, cells were harvested from microcarriers using TrypLE™ (Invitrogen, Carlsbad CA) and cryopreserved and stored in liquid nitrogen. Cells were stored until labeling with 111-Indium (111-In) oxine. While 111-In labeling was not required for the MRI analysis described in herein, it allowed for cell tracking to be performed in the same animals using *in vivo* single photon emission computed tomography (SPECT) imaging (Arbab. *et al*; submitted).

### Animal Model of Middle Cerebral Artery Occlusion

Male Wistar rats (n = 21) weighing 270∼300 g were subjected to 2 hours intraluminal transient middle cerebral artery occlusion (tMCAo), as previously described [45], and randomized into two groups: vehicle (serum-free proprietary formulation) only (control group, N = 10), or 0.5 mCi 111-In labeled hUTC (treated group, N = 11) 48 h after MCAo. During surgery and injection of vehicle or 111-In labeled hUTC, animals were anesthetized with 3.5% isoflurane and then maintained with 1.0∼2.0% isoflurane in N_2_O:O_2_ (2∶1). Approximately, 3×10^6^ 111-In labeled hUTC in 2 ml total fluid volume of serum-free proprietary formulation were injected intravenously via the tail vein.

### MRI Measurements

MRI measurements were performed using a 7T, 20 cm bore superconducting magnet (Magnex Scientific, Abingdon UK). A 12 cm bore actively shielded gradient coil set, capable of producing magnetic field gradients up to 20 gauss/cm was used. A saddle radio-frequency (RF) coil was used as the transmitter and a surface coil as the receiver. Stereotaxic alignment system equipped with ear bars were used to minimize head movement during the imaging procedure. During MRI measurements, anesthesia was maintained using a gas mixture of N_2_O (69%), O_2_ (30%) and isoflurane (1–1.5%). Rectal temperature was kept at 37±0.5°C using a feedback controlled heat pad. A tri-pilot scan of imaging sequence was used to position head of the animal with the central image slice located at the level of the bregma [46]. MRI measurements including: diffusion tensor (fractional anisotropy, FA), permeability (blood-to-brain transfer constant, Ki), and perfusion (cerebral blood volume, CBV, and cerebral blood flow, CBF), T_2_, and T_2_* were performed at one day before treatment (one day after onset of stroke), and 2, 3, 6, and 12 weeks after stroke. *Ex vivo* Q-space DTI was performed after sacrifice at 12 weeks after stroke.

### Diffusion Tensor MRI Measurement

Diffusion Tensor MRI was acquired using Stejskal-Tanner pulsed gradient spin-echo sequence with 32 mm field of view (FOV), 128×128 imaging matrix, 1 mm slice thickness with 13 slices, TR = 1.5 sec and TE = 40 msec, six diffusion weighted images along six independent axes with b = 0 and 900 sec/mm^2^ on each slice. The total acquisition time was approximately 22.5 min.

### T_2_ Measurement

T_2_ was measured using a Carr-Purcell-Meiboom-Gill multislice multiecho (six echo) MRI. A series of four sets of images (13 slices for each set) were obtained using TEs of 15, 30, 45, 60, 75, and 90 msec and a TR of 4 sec. Images were produced using a 32 mm FOV, 1 mm slice thickness, 128×128 image matrix. The total time for the entire sequence was approximately 8.5 min.

### T_2_* Measurement

T_2_* was obtained using a gradient echo multislice multiecho sequence. A TR value of 4 sec was used with echo times of 3.5, 7, 10.5, 14, 17.5, and 21 msec. Images were produced with a 32 mm FOV, 1 mm slice thickness, 13 slices, and 128×128 image matrix. The total scan time was 9 min for the complete image data set.

### Look Locker (L-L) T_1_ Measurement

An imaging variant of the Look-Locker technique [7], [47] employing the T-One by Multiple Read-Out Pulses (TOMROP) [48] sequence was used for a pixel-by-pixel estimate of T_1_. An initial inversion of magnetization, followed by a set of small-tip-angle gradient-echo readouts and a waiting period for the magnetization to recover toward equilibrium, results in a set of images that approached a steady-state magnetization with a time constant, T_1_*. T_1_ was calculated from its relationship with T_1_* as, 1/T_1_* = 1/T_1_ - ln (cosα)/?, where α is the readout pulse flip angle and ? is the inter-readout-pulse interval. Interleaved slices were acquired using a numerically optimized RF pulse [49]. Inversion was accomplished using a non-selective hyperbolic secant adiabatic pulse of 12 msec duration. One phase-encoded line of 24 small-tip-angle (α = 18°) gradient-echo images (TE 4 msec) was acquired at ϑ = 50****msec intervals after each such adiabatic inversion for a total recovery time of 1200 msec with a 3 sec relaxation interval between each inversion. Matrix size was 128×64, FOV 32 mm, and five 2 mm slices were imaged.

### Vascular Permeability Related MRI Measurement

To measure permeability related parameters, five slices L-L measurements were used to acquire dynamic R_1_ (1/T_1_) maps [7], [50]. After the acquisition of one R_1_ map, a bolus of 0.2 mmol/kg Gd-DTPA injection via the tail vein was following by a 0.4 ml saline flush. After the Gd-DTPA bolus injection, 12 R_1_ maps were then acquired. Time-dependent changes in R_1_ was used to form a map of the blood-to-brain transfer constant (K_i_) and the distribution volume of protons relaxed by the contrast agent (V_p_). This is accomplished *via* a model-independent analysis utilizing a Patlak plot [7], [50].

### *Ex-Vivo* Q-Space DTI MRI Measurement

Q ball based diffusion tensor imaging (DTI) was acquired using Stejskal-Tanner pulsed gradient spin-echo sequence. The field of view was 32 mm; two averages were taken using 128×128 imaging matrix, 1 mm slice thickness with 13 slices, TR = 1.5 sec and TE = 40 msec, δ = 10 msec, Δ = 18 msec, 128 diffusion directions with b = 1200 sec/mm^2^ and six baseline images with b = 0 on each slice.

### Neurobehavioral Testing

The modified neurological severity score (mNSS) [41], foot-fault [51], and adhesive removal [41], [52] behavioral tests were performed before tMCAo (data not shown), 1 day after tMCAo (1 day before treatment), and weekly thereafter by an investigator who was blinded to the identity of the experimental groups. The size of the adhesive tab was reduced to a half size starting at 2 months (or 56 days) post-tMCAo in order to increase the sensitivity of the adhesive-removal test measurement.

### Brain Section Preparation and Immunohistochemistry Evaluation

Rats were transcardially perfused with heparinized saline and brains were rapidly removed after final MRI measurements at 12 weeks after stroke. Brain section preparation, infarct volume, synaptophysin or vWF immunoreactivity for synaptic and microvessel densities respectively, Bielschowsky and luxol fast blue staining for axons and myelin and terminal deoxynucleotidyl transferase (TdT)–mediated dUTP-biotin nick end labeling (TUNEL) method for apoptotic cell measurements were performed as previously described [6], [7], [41], [53], [54].

### Immunohistochemistry Data Analysis

The number of apoptotic cells per section was counted in the ipsilateral hemisphere. For measurement of vascular and synapse density, 8 FOV from the ischemic boundary zone (IBZ) were digitized using a 40X objective and the MCID system [11] ^, 37^. The number of vessels/mm^2^ and synaptophysin (percent area) were measured in the IBZ throughout each FOV and the enlarged vascular perimeter was reported as the total perimeter of 10 enlarged vessels in the ipsilateral hemisphere. For quantification of myelinated axons, Bielschowsky and luxol fast blue positive areas were digitized throughout the ischemic boundary zone, as well as the contralateral homologous area. Data are presented as the percentage of Bielschowsky and luxol fast blue positive area compared with the contralateral homologous region on the same section.

### MRI Data Processing and Measurements

T_2_, T_2_*, CBF, CBV, K_i_, and FA were calculated on a pixel by pixel basis [6], [7], [55]. The *ex vivo* Q-space DTI data was analyzed using our in-house software to derive diffusion standard deviation (SD) map [56] and using Camino for Q-space fiber tracking [57], [58]. The SD map was created based on calculating the deviation of diffusivity from a sphere for each voxel in the image. For this calculation all 55 measurements were used. If the voxel falls in an ideal isotropic medium, diffusion will also be isotropic in all directions. In such a case, the diffusion vectors will form a sphere and the standard deviation of the length of these vectors will be zero. If for any reason, the envelope of these vectors forms any other shape, the standard deviation will have a non-zero value. Therefore, if diffusion is constrained by tubular structures, the SD of the length of the vectors will possess a non-zero value based on the complexity of the structure.

MRI ischemic lesion was determined on T_2_ map. The lesion area on each slice of T_2_ map was specified by those pixels with a T_2_ value higher than the mean plus twice the standard deviation (mean +2SD) measurements provided by the normal tissue on the contralateral side. Lesion volume was obtained by adding all the areas measured on individual slices and multiplying each area by the slice thickness.

The volume of the lateral ventricle was measured on T_2_ maps at a fixed structural location presented by six contiguous coronal T_2_ slices using the same criteria as described above to identify the ventricular area on each slice. The ventricular volume was obtained by adding all the areas measured on individual slices and multiplying each area by the slice thickness. The ventricle volumes measured at various time points were normalized for each animal to the ventricular volumes measured at 24 hrs after tMCAo and 24 hrs before treatment to reduce errors from individual variations.

**Figure 6:**
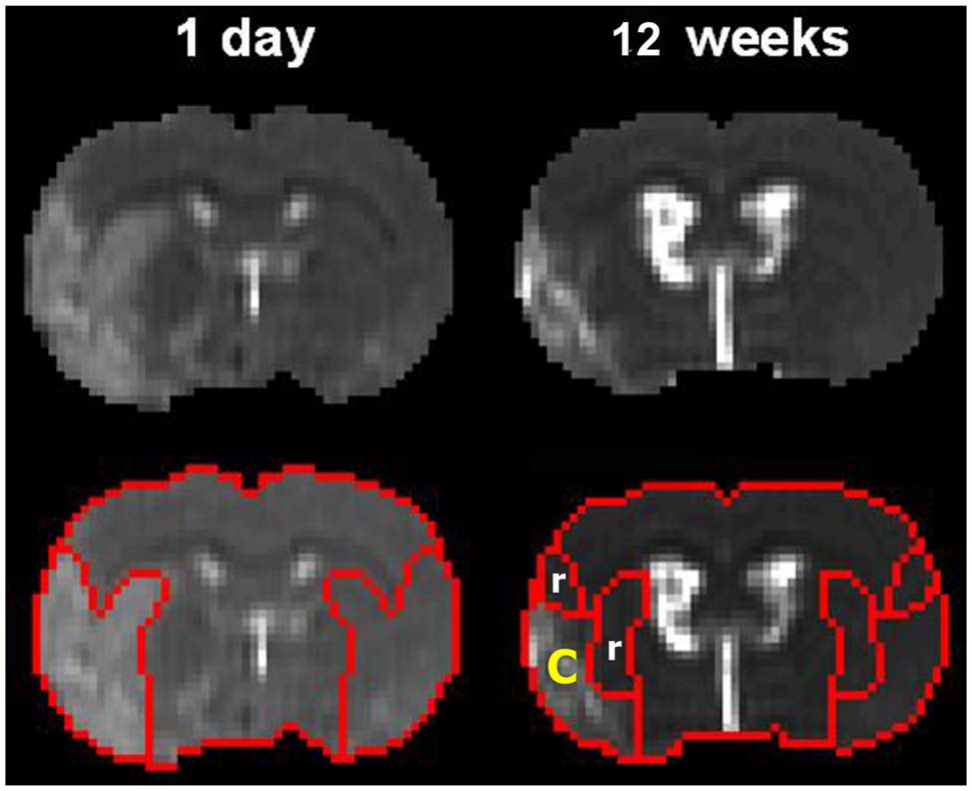
Diagrams of the ischemic ROI. Diagrams of the ischemic core (c, 12 weeks) and ischemic recovery (r, 12 weeks) regions of interest (ROI) determined from T2 maps. FA (A), T_2_ (B), T_1_ (C), and T_1sat_ were measured from these ROIs.

MRI regions of interest (ROI) were identified in the ischemic core and recovery areas, and in a homologous location in the contralateral hemisphere from T2 maps (Fig. 6). Two ROIs were selected for analysis of MRI parameters. The first ROI, the ischemic core area, was identified by using the threshold T_2_ value of mean + two standard deviations from the T_2_ value measured in the contralateral hemisphere on T_2_ maps obtained 12 weeks after stroke. The ischemic areas in T_2_ maps were always smaller at 12 weeks after stroke compared to that at 24 hrs after stroke. Therefore, the second ROI, the ischemic recovery area, was identified by subtracting the ischemic core areas obtained 12 weeks after stroke from the ischemic area in T_2_ maps obtained 24 hrs after stroke. Homologous ROIs to the ischemic core and recovery tissues were also measured in the contralateral hemisphere. All MRI parameters were measured from those two regions.

### Statistical Analysis

This study investigated the effect of hUTC on the functional recovery, measured from three behavioral tests over time after tMCAo, as well as the MRI alterations and histological measurements. Data were evaluated for normality. Ranked data was used in the analysis for functional tests, since those data were not normally distributed. The global test, using Generalize Estimating Equation (GEE), was implemented to evaluate the functional recovery measured from three behavioral tests, repeated over time. Data analysis began by testing the interaction between treatment and time, followed by testing the main effect of treatment or time on the functional recovery if no interaction was detected at the 0.05 level. A significant treatment-by-time interaction (p<0.05) would indicate that the effects of the hUTC treatment (compared to vehicle controls) might depend on the time of assessments, or the effects of time might depend on treatment groups. Subgroup analysis (CONTRAST statement in SAS) was then used to test hUTC effect compared to vehicle treated group over time, or test the time effect at each treatment group [59].

A linear regression model was used to test differences in the MRI parameters between the ischemic core and recovery areas, adjusted for the treatment group and time of MRI acquisition. The mixed model, analysis of variance and covariance (ANCOVA), was used to study treatment group effect or time effect on the MRI measurements at each ROI (ischemic core or recovery area), respectively. The analysis approach was similar to what was described above for functional recovery. Analysis began by testing for the interaction between the treatment and time followed by subgroup analysis. Figures showed mean ± SD changes over time for each treatment groups.

The relationship between MRI parameters and functional recovery, as well as the immunohistological measurements, were determined by Pearson/Spearman correlation coefficients.
